# Post-COVID-19 Changes in Appetite—An Exploratory Study

**DOI:** 10.3390/nu16142349

**Published:** 2024-07-20

**Authors:** Georgeta Inceu, Ruben Emanuel Nechifor, Adriana Rusu, Dana Mihaela Ciobanu, Nicu Catalin Draghici, Raluca Maria Pop, Anca Elena Craciun, Mihai Porojan, Matei Negrut, Gabriela Roman, Adriana Fodor, Cornelia Bala

**Affiliations:** 1 Department of Diabetes and Nutrition Diseases, “Iuliu Hatieganu” University of Medicine and Pharmacy, 400006 Cluj-Napoca, Romania; inceu.victoria@umfcluj.ro (G.I.); adriana.rusu@umfcluj.ro (A.R.); anca.craciun@umfcluj.ro (A.E.C.); groman@umfcluj.ro (G.R.); adriana.fodor@umfcluj.ro (A.F.); cbala@umfcluj.ro (C.B.); 2 Department of Diabetes, Emergency Clinical County Hospital Cluj, 400006 Cluj-Napoca, Romania; 3 International Institute for the Advanced Studies of Psychotherapy and Applied Mental Health Department of Clinical Psychology, Psychotherapy Babes-Bolyai University, 400294 Cluj-Napoca, Romania; ruben.nechifor@ubbcluj.ro; 4 Department of Clinical Neurosciences, “Iuliu Hațieganu” University of Medicine and Pharmacy, 400012 Cluj-Napoca, Romania; nicu.draghici@umfcluj.ro; 5 “IMOGEN” Institute, Centre of Advanced Research Studies, Emergency Clinical County Hospital Cluj, 400012 Cluj-Napoca, Romania; 6 Department of Morphofunctional Sciences, Pharmacology, Toxicology and Clinical Pharmacology, “Iuliu Hatieganu” University of Medicine and Pharmacy, 400012 Cluj-Napoca, Romania; parlograluca@gmail.com; 7 Department of Internal Medicine, “Iuliu Hatieganu” University of Medicine and Pharmacy, 400012 Cluj-Napoca, Romania; porojan78@yahoo.com; 8 Department of Internal Medicine, Emergency Clinical County Hospital Cluj, 400012 Cluj-Napoca, Romania; 9 Faculty of Medicine, “Iuliu Hatieganu” University of Medicine and Pharmacy, 400012 Cluj-Napoca, Romania; mateinegrut@gmail.com

**Keywords:** appetite, COVID-19, fMRI, ghrelin, neuropeptide Y

## Abstract

In this analysis, we aimed to investigate the effect of COVID-19 disease on eating behavior. A total of 55 right-handed adults, <50 years of age, without overweight or obesity, from two cross-sectional studies were included. The first one enrolled subjects between September 2018 and December 2019 (non-COVID-19 group). The second one included subjects enrolled between March 2022 and May 2023; for this analysis, 28 with a history of COVID-19 (COVID-19 group) were retained. Hunger, TFEQ-18, plasma ghrelin, neuropeptide Y (NPY) and resting-state fMRI were assessed during fasting. Intraregional neuronal synchronicity and connectivity were assessed by voxel-based regional homogeneity (ReHo) and degree of centrality (DC). Significantly higher ghrelin and NPY levels were observed in the COVID-19 group than in the non-COVID-19 group (ghrelin 197.5 pg/mL vs. 67.1 pg/mL, *p* < 0.001; NPY 128.0 pg/mL vs. 84.5 pg/mL, *p* = 0.005). The NPY levels positively correlated with the DC and ReHo in the left lingual (r = 0.67785 and r = 0.73604, respectively). Similar scores were noted for cognitive restraint, uncontrolled eating and emotional eating in both groups according to the TFEQ-18 questionnaire results (*p* > 0.05 for all). Our data showed increased levels of appetite-related hormones, correlated with activity in brain regions involved in appetite regulation, persisting long after COVID-19 infection.

## 1. Introduction

The COVID-19 pandemic has affected millions of lives worldwide, generating an unprecedented global health, economic, social, and humanitarian crisis [1]. While primarily characterized by respiratory symptoms, mounting evidence confirms that COVID-19 manifests with a diverse array of short- and long-term systemic effects, leading to multiple-organ dysfunction caused by the overproduction of pro-inflammatory chemokines and cytokines [2,3,4]. 

One of the systemic consequences of COVID-19 infection is believed to be alterations in appetite regulation [5], a complex process involving intricate interactions between peripheral signals and central neural circuits [6]. Among the various hormones implicated in appetite modulation, ghrelin, produced in the oxyntic glands of the gastric fundus, plays a pivotal role. Ghrelin not only stimulates appetite but also influences energy homeostasis, reward processing and metabolic function, and it plays an important role in gastric motility, gastric acid secretion, immune functions, and cell proliferation as well as heart function [7,8]. Its multifaceted actions make it a key player in the intricate network governing food intake and energy balance. 

A recent study showed a significantly higher concentration of ghrelin in the group of patients with COVID-19 up to six months post-infection compared to healthy people of the same age and comparable body mass index (BMI) [9], a finding that may suggest an association between ghrelin and the immune system. Consistent data from the literature have shown that ghrelin exerts anti-inflammatory effects, indicating the role of this hormone as a potential treatment for inflammatory conditions [10,11]. Yorulmaz et al. [12] have demonstrated that ghrelin treatment improved the severity of lung tissue injury during sepsis in rats, reducing the level of pro-inflammatory cytokines and increasing the antioxidant effect of superoxide dismutase enzyme. 

One study analyzed the influence of the COVID-19 virus on the level of ghrelin, aiming to determine the influence of this hormone on appetite, but the results showed no significant differences in the level of serum ghrelin in acute COVID-19 patients compared with healthy controls [13]. 

The objective of this study was to investigate the effect of COVID-19 disease on eating behavior, hunger, and cognitive control of eating in patients with a COVID-19 history compared with a control group. 

## 2. Materials and Methods

### 2.1. Study Design and Study Subjects

Data from 2 cross-sectional observational studies performed in the Diabetes and Nutrition Diseases Department of “Iuliu Hatieganu” University of Medicine and Pharmacy Cluj-Napoca, Romania, were included in this analysis. The first dataset included right-handed healthy subjects enrolled between September 2018 and December 2019 (before the COVID-19 pandemic, non-COVID-19 group) and fulfilling the following inclusion criteria: BMI < 25 kg/m^2^, aged between 18 and 50 years, average daily caffeine consumption ≤350 mg and alcohol consumption <6 units/week. We chose these inclusion criteria to exclude changes in appetite previously reported to be associated with overweight and increased alcohol and caffeine consumption [14,15,16]. The 2nd dataset included right-handed subjects enrolled between March 2022 and May 2023, aged 18 to 50 years, with an average daily caffeine consumption of ≤350 mg and alcohol consumption of <7 units/week for women and <14 units/week for men, without a history of diabetes, without any medication that would interfere with appetite at the time of enrollment (e.g., GLP-1 receptor agonists, corticosteroids), or a history of drug abuse or recreational drug use within the 4 weeks before study inclusion. For the current analysis, from this 2nd dataset, only subjects with a history of COVID-19 were selected for study inclusion (COVID-19 group). Subjects with an acute disease within 2 weeks before study inclusion, those working in shifts, subjects with a history of psychiatric disorders at enrolment (including anxiety, depression, eating disorders or disordered eating behaviors like anorexia, bulimia, nocturnal eating), and pregnant and lactating women were excluded from both studies. 

The study protocols, information sheets and informed consent forms were approved by the Ethics Committees of the “Iuliu Hatieganu” University of Medicine and Pharmacy Cluj-Napoca, Romania (Ethics Committee approval numbers 23/7 February 2022 and 242/31 May 2018). Both research projects were conducted according to the principles of the Declaration of Helsinki of the World Medical Association, relating to the conduct of ethical research on human beings and local regulations. All the participants were informed about the nature of the study, personal data collection and use, and signed the informed consent form before any study-related procedures. 

### 2.2. Assessments and Data Collection

In both studies, subjects underwent 2 study visits performed in a fasting condition. Data on demographic parameters (age, gender, education, employment status), medical history, medication use, lifestyle (habitual caffeine consumption, weekly alcohol consumption, smoking status, eating behavior and average time spent being sedentary/day), and hunger were collected through interviews and questionnaires. Anthropometric parameters (weight, height, and waist circumference) and blood pressure were measured using calibrated scales and a sphygmomanometer. Blood samples were collected for the assessment of glycemia, serum aspartate and alanine aminotransferases and creatinine levels in the Emergency Clinical County Hospital Cluj laboratory by commercially available routine enzymatic methods (Beckman-Coulter AU680, Beckman Coulter Inc., Brea, CA, USA) on the day of the sample collection. Psychiatric disorders were excluded by medical history, questionnaires (General Anxiety Disorder Scale (GAD7) [17], Patient Health Questionnaire (PHQ9) [18]), and interviews performed by a certified clinical psychologist in cases with high GAD-7 or PHQ9 questionnaire score. The GAD-7 and PHQ9 were translated and validated in the Romanian language and their psychometric properties were reported elsewhere [19,20] (https://www.phqscreeners.com/, accessed on 15 April 2024). For the sample included in this analysis, the GAD-7 and PHQ9 had good reliability, with Cronbach’s alpha values of 0.846 and 0.646, respectively. fMRI image acquisition was performed on a different day, in the morning before 10:00 a.m., under fasting conditions, with subjects lying awake, with eyes closed and without concentrating their thoughts on anything specific [21]. 

### 2.3. Hunger, Desire to Eat, Fullness Sensation and Eating Behavior Assessment

Hunger, desire to eat and fullness sensation were assessed in the fasting state by 100 mm visual analogue scales (ranging from 0 for “not at all” to 100 for “very much”) previously described by Flint et al. [22]. Blood samples were collected using EDTA tubes after at least 8 h of fasting, before 10:00 a.m., and before appetite and hunger evaluation. Samples were centrifuged at 1000× *g* for 10 min within 30 min after collection, and plasma was aliquoted and stored at −80 °C until assessment. The ELISA protocols were performed according to the manufacturer’s guidelines using commercially available ELISA kits (Elabscience Bionovation Inc, Houston, Texas) in two series for acylated ghrelin (code: E-EL-H2002) and one series for neuropeptide Y (NPY) (code: E-EL-H1893). During analysis, the non-specific material was removed from the wells using the BioTek Microplate 50 TS washer (Agilent Technologies Inc., Santa Clara, CA, USA). The plate absorbance was read using the 800 TS ELISA microplate reader (Agilent Technologies Inc., Santa Clara, CA, USA). Additionally, standard curves were plotted, and data were calculated using the software of the plate reader (Gen5 3.11).

For the eating behavior assessment, we used questions on the number of meals/day, eating breakfast daily or on most of the days, the main meal of the day, eating dinner after 21:00, and eating while watching TV/playing computer/reading [23]. Cognitive restraint, uncontrolled eating, and emotional eating were investigated using the Three-Factor Eating Questionnaire-18 (TFEQ-18) version 2 [24] (Cronbach’s alpha of 0.862 for the sample included in this analysis).

### 2.4. fMRI Image Acquisition, Preprocessing and Analysis

The MAGNETOM 3T Skyra (SIEMENS, Munich, Germany) with 20 channels head coil and the Discovery MR750W 3.0T (General Electric) with a 3.0T GEM HNU head coil were used for the structural T1 MPRAGE sequence and for the functional BOLD sequence acquisition. As previously described [21], the following parameters were used for the structural volumetric T1 MPRAGE image acquisition: an echo spacing of 6.4 ms, bandwidth of 220 Hz/Px, 160 slices per slab, TR of 1.9 s. An FOV of 230 mm and voxel size of 0.4 × 0.4 × 1 mm. The T1 MRI data had a total acquisition time of 4 min and 22 s. For the resting-state BOLD images, the acquisition parameters were as follows: echo spacing = 0.65 ms, bandwidth = 1776 Hz/Px, 180 volumes (interleaved slices/volume), slice thickness = 3 mm; matrix = 4.4 × 4.4 × 3 mm, TR = 2 s, FOV = 280 mm, acquisition time = 6 min and 6 s. The GRAPPA acceleration mode (acceleration factor PE = 2 and ref. lines PE = 24) was used for faster structural and functional acquisitions [21]. 

The resting-state fMRI images were preprocessed using the Data Processing Assistant for Resting-State fMRI (DPARSF version 4.5) [25]. Preprocessing included the removal of the first 10 volumes to allow for signal equilibration, spatial realignment for head motion correction within and across fMRI runs, nuisance covariates regression (regressing out head motion parameters, white matter and cerebrospinal fluid signals in the whole brain) using the Friston 24 model [26,27], normalization to the Montreal Neurological Institute (MNI) space by exponentiated Lie Algebra (DARTEL) algorithm [28] and temporally band-pass filtering to retain frequencies in the 0.01 < f < 0.1 Hz band. To further reduce the influence of residual motion-related abnormalities in the data, scrubbing was applied using the framewise displacement (FD) Jenkinson approach [29] and frames with FD ≥ 0.2 mm were removed. Finally, the T1 structural images were co-registered to the functional space, segmented in gray matter, white matter and cerebrospinal fluid and normalized to the MNI space by diffeomorphic anatomical registration by DARTEL [28]. All the participants had a maximum head motion <1.5 mm and <1.5 degree and thus all were included in the analysis. 

The first-level analysis was also performed in DPARSF. To evaluate the resting-state brain functional synchronization, regional homogeneity (ReHo) and degree of centrality (DC) maps of each subject were calculated based on the pre-processed images. The analysis of ReHo and DC was restricted to a gray matter mask generated during the preprocessing step. At the last step, the ReHo and DC maps were spatially smoothed using a Gaussian kernel (8 mm full width at half-maximum [FWHM] isotropic). ReHo and DC are measures of brain resting-state network connectivity. ReHo evaluates temporal synchronization within the nearest neighbors of a given voxel [30], while the DC allows the assessment of connectivity between a brain region and the whole brain network at the voxel level [31].

### 2.5. Statistical Analysis

This was an exploratory post hoc analysis, meaning no sample size calculation was performed and a convenience sample of subjects per study group was chosen. Statistical analysis was performed with IBM SPSS Statistics for Windows, Version 26.0 (IBM Corp, Armonk, NY, USA). Data were presented as the median (quartile 1; quartile 3) for continuous variables and number (percentage) for categorical variables. The COVID-19 and non-COVID-19 study groups were compared using Mann–Whitney U and chi-square tests according to the type of variables included. 

The correlations of appetite-related data with the ReHo and DC statistical maps were performed in the Data Processing and Analysis of Brain Imaging (DPABI) version 4.2 [32]. The correlation results were adjusted for multiple comparisons using a two-tailed Gaussian Random Field (GRF) correction with a statistical significance threshold set to a voxel *p*-value < 0.001 and a cluster *p*-value < 0.05 and were masked with the mean gray matter image of all the participants. Through these procedures, we identified ReHo and DC clusters correlated to the appetite variables. The anatomical location of each cluster was identified using the MNI coordinates of the peak voxel in the cluster and atlases provided by DPABI (automated anatomical labeling and Brodmann). The peak T values depict correlation coefficients, with positive ones showing positive correlations and negative ones showing negative correlations between the ReHo, DC and appetite-related parameters.

## 3. Results

### 3.1. Demographic and Clinical Characteristics

A total of 55 adults fulfilling the inclusion criteria and without any exclusion criterion were included in this analysis (28 with a COVID-19 history before study inclusion and 27 without). None had overweight, obesity, diabetes, coronary heart disease, stroke history or psychiatric disease. 

The general characteristics of the COVID-19 and non-COVID-19 groups are presented in Table 1. There were no statistically significant differences in the anthropometric and clinical parameters between the groups. However, a statistically significant difference can be observed between the groups regarding the total time spent being sedentary/day (8.3 h/day in the COVID-19 group vs. 6.0 h/day in the non-COVID-19 group, *p* = 0.035) and fasting glycemia and ALAT values (*p* < 0.05 for both). In the COVID-19 group, the median time since the last COVID-19 infection was 13.0 months (min 1 month; max 27 months).

### 3.2. Eating Behavior and Appetite 

No statistically significant differences between the groups were observed in terms of eating behavior (Table 2). A similar percentage of participants declared eating three meals/day daily or on most of the days, having breakfast daily and eating dinner after 21:00 (*p*-value for all >0.05). The frequency of eating while watching TV was numerically higher in the COVID-19 group (67.9%) compared to the non-COVID-19 group (55.6%), but the difference did not reach statistical significance.

Significantly higher ghrelin and NPY levels in the fasting condition were observed in the COVID-19 group than in the non-COVID-19 group (197.5 pg/mL vs. 67.1 pg/mL, *p* < 0.001 for ghrelin and 128.0 pg/mL vs. 84.5 pg/mL, *p* = 0.005; Table 3). No difference was observed in the rating of self-perceived hunger, fullness, and desire to eat under fasting conditions. Also, similar scores were noted for cognitive restraint, uncontrolled eating and emotional eating in both groups according to the TFEQ-18 questionnaire (*p* > 0.05 for all). 

### 3.3. Correlation Analysis of Appetite-Related Data with Resting-State fMRI

**Figure 1:**
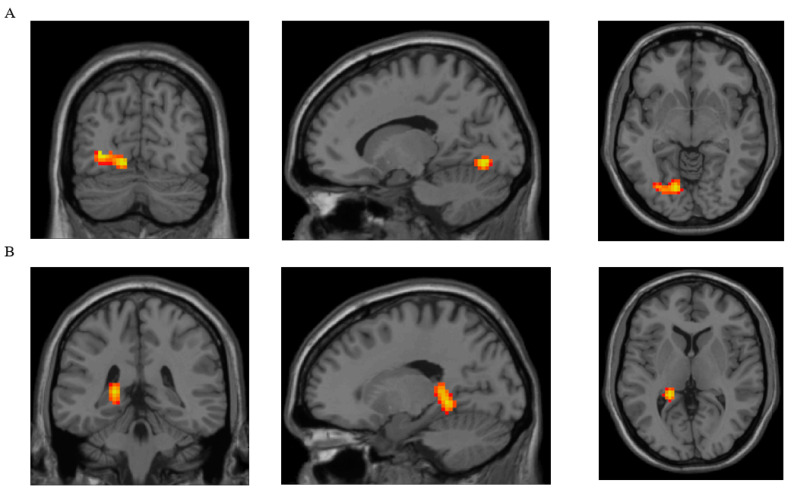
Brain regions showing significant correlations with NPY. (**A**). DC of left lingual (BA 18 L) was positively correlated with NPY value. (**B**). ReHo of left lingual (BA 37 L) was positively correlated with NPY value. NPY = neuropeptide Y; DC = degree of centrality; ReHo = regional homogeneity.

The appetite-related data were further tested for correlations with the resting-state intraregional neuronal synchronicity and connectivity, as assessed by the voxel-based ReHo and DC statistical maps. Correlation analysis showed a positive correlation of the NPY levels with the DC in the left lingual, Brodmann area 18 L (r = 0.67785). Also, a positive correlation of the NPY levels was observed with the ReHo in the left lingual, Brodmann area 37 L (r = 0.73604). The resting-state activity in the brain regions that were significantly correlated with NPY are presented in Table 4 and Figure 1. No correlations of hunger, fullness sensation, desire to eat, cognitive restraint, uncontrolled eating, emotional eating, and ghrelin with ReHo and DC were observed (no cluster survived the GFR correction threshold).

## 4. Discussion

The main findings of our research were the persistent higher acylated ghrelin and NPY plasma levels in subjects with a history of COVID-19 infection as compared to those before the COVID-19 pandemic, albeit without any difference in self-rated fasting hunger, fullness sensation and desire to eat. 

Scarce data on the influence of COVID-19 on appetite-regulating hormones are available; to the best of our knowledge, only two studies on the ghrelin levels after COVID-19 have been published so far [9,13]. One study analyzed the potential effect of the COVID-19 virus on the level of blood ghrelin to determine the impact of this infection on the appetite. The results showed no significant changes in the serum ghrelin level in COVID-19 patients [13]. Our results were similar to those published by Kuliczkowska-Płaksej [9]. In a study aiming to assess the appetite-related hormone levels 6 months after COVID-19, the authors found higher ghrelin levels in patients with a COVID-19 history compared to healthy persons matched for age and BMI. Furthermore, our results show the persistence of higher ghrelin levels years after the COVID-19 disease.

Ghrelin is a multifunctional hormone with diverse physiological effects beyond its well-known role in appetite regulation. The physiological functions of ghrelin have been extensively studied in the last few years. Ghrelin is a peptide hormone primarily produced by the stomach, although it is also synthesized in other tissues, including the brain. It plays a crucial role in regulating appetite and energy balance by promoting food intake [33]. Additionally, ghrelin affects reward pathways in the brain, increasing the pleasure associated with eating [34]. Beyond regulating appetite and energy expenditure, ghrelin serves other several physiological functions, including glucose metabolism regulation [35], gastrointestinal motility and acid secretion [36], and cardiovascular function [37], and it may play a role in cognitive function and mood regulation [38]. Also, recent research has suggested that ghrelin may have implications in relation to inflammation and the immune system, although the exact mechanisms are still being investigated. Several studies have indicated that ghrelin exhibits anti-inflammatory properties. It may suppress the production of pro-inflammatory cytokines, such as tumor necrosis factor-alpha (TNF-alpha), interleukin-6 (IL-6), and interleukin-1 beta (IL-1β), while promoting the release of anti-inflammatory cytokines like interleukin-10 (IL-10) [8,39]. Ghrelin receptors are expressed on various immune cells, including monocytes, macrophages, and T lymphocytes. Activation of these receptors may influence immune cell function and cytokine production, thereby regulating immune responses [40]. Since ghrelin has been shown to possess anti-inflammatory properties, it is plausible that its levels could be affected by the inflammatory response triggered by COVID-19. 

An important finding of our research was the higher NPY plasma levels in persons with a history of COVID-19 infection within the 2 years prior to study inclusion as compared to those before the COVID-19 pandemic. NYP, a 36 amino acid peptide that belongs to the pancreatic polypeptide family, is one of the most abundant peptides found in the brain but can also be present in the peripheral nervous system [41]. It is considered to be the most potent orexigenic hormone in the brain, but NPY is associated with diverse biological actions, including cortical excitability, stress response, cardiovascular regulation, seizure and cognition, and appetite regulation [42]. The biological actions of NPY are mediated by six receptors, Y1–Y6, but the effects on feeding are mediated through at least two receptors, the Y1 and Y5 receptors [43]. While there is currently limited direct evidence of the influence of COVID-19 on NPY levels, the virus could potentially impact NPY through various mechanisms related to stress, inflammation, metabolism, and complications associated with infection. 

Ghrelin and neuropeptide Y (NPY) are both key regulators of appetite and energy balance, and they interact within the central nervous system to modulate feeding behavior and metabolic processes. While they operate through distinct pathways, there are several points of correlation and interaction between ghrelin and NPY, including central regulation of feeding behavior, interactions in the hypothalamus, modulation of neurotransmitter release and integration with other hormonal signals [44]. 

Our findings regarding higher appetite-related hormones are supported by the positive correlation found between the NPY levels and the resting-state brain activity in the left lingual cortex, Brodmann areas 18 and 37, implying higher activity in the lingual gyrus in those with higher NPY levels. The lingual gyrus is part of the visual association cortex, and its activation is modulated by the emotional salience of the stimuli, thus being involved in the visual processing of food cues [45]. A study performed in healthy adults showed that intragastric and intravenous infusion of glucose induced a decrease in the activity of the left lingual gyrus and the activity temporally correlated with insulin and GLP-1 levels, supporting its participation in gut-to-brain signaling pathways [46]. A meta-analysis of task-based fMRI studies found activation of the left lingual gyrus in response to food pictures in healthy normal weight adults [45]. Also, changes in food craving (nonfood vs. food cues) have been associated with left lingual gyrus activation in women with obesity [47].

There are some limitations of our study. One of these limitations refers to the fact that cross-sectional does not imply causality. Also, the small sample size is another important limitation, which may have influenced the statistical significance of the results. The subjects included in this study were evaluated at different time intervals after the COVID-19 infection, with a potential impact on variables assessed. Another important limitation is that we were not able to perform a before- and after-COVID-19 study. Although the COVID-19 and control groups included only normal-weight healthy subjects and were well matched in terms of age, BMI, and other clinical characteristics, we cannot preclude an influence of individual factors on the results.

## 5. Conclusions

In conclusion, our data showed increased levels of appetite-related hormones correlated with activity in brain regions involved in appetite regulation persisting long after COVID-19 infection in healthy subjects without overweight or obesity. Despite the study limitations, these results suggest potentially long-term consequences of COVID-19 infection for weight, independent of lifestyle changes induced by lockdown during the pandemic. To confirm our findings and further describe the effect of COVID-19 on appetite, larger-sample prospective studies with serial evaluation of appetite hormones are needed.

## Figures and Tables

**Table 1:** Participants’ characteristics according to COVID-19 status.

	COVID-19 N = 28	Non-COVID-19 N = 27	*p*-Value
Women, n (%)	18 (64.3%)	19 (70.4%)	0.631
Men, n (%)	10 (35.7%)	8 (29.6%)	
Age, years	28.0 (26.0; 29.0)	29.0 (27.0; 34.5)	0.140
University education, n (%)	25 (92.6%)	27 (100.0%)	0.150
Hypertension, n (%)	1 (3.6%)	0	-
Never smokers, n (%)	21 (75.0%)	21 (18.577.8%)	0.579
Alcohol portions/week	0.3 (0.0; 1.5)	0.0 (0.0; 2.0)	-
BMI, kg/m^2^	18.4 (16.9; 21.6)	18.1 (16.2; 20.3)	0.391
Waist circumference, cm	72.5 (70.0; 90.5)	76.0 (70.0; 82.0)	0.980
SBP, mmHg	119.5 (101.3; 129.0)	111.0 (100.0; 123.0)	0.192
DBP, mmHg	72.5 (68.0; 82.0)	72.0 (66.5; 79.5)	0.866
Fasting glycemia, mg/dL	77.0 (72.0; 82.5)	82.5 (78.0; 86.0)	0.037
AST, UI/L	20.5 (18.0; 26.0)	19.5 (17.0; 23.5)	0.627
ALT, UI/L	19.5 (13.0; 31.3)	13.5 (10.8; 21.5)	0.044
Creatinine, mg/dL	0.8 (0.7; 0.9)	0.7 (0.6; 0.8)	0.143
Total time spent being sedentary/day, hours	8.3 (5.5; 10.0)	6.0 (4.3; 8.0)	0.035
GAD score	2.0 (1.0; 4.0)	3.0 (1.5; 4.0)	0.798
PHQ-9 score	3.0 (1.0; 4.0)	2.0 (2.0; 6.0)	0.458
Time since the last COVID-19 episode, months	13.0 (11.0; 15.0)	-	-

N/n (%) = number (percentage) of participants; AST = aspartate aminotransferase; ALT = alanine aminotransferase; GAD = Generalized Anxiety Disorder; PHQ-9 = Patient Health Questionnaire-9; BMI = body mass index; SBP = systolic blood pressure; DBP = diastolic blood pressure. Data in the table are provided as the median (quartile 1; quartile 3), unless otherwise specified.

**Table 2:** Eating behavior according to COVID-19 status.

	COVID-19 N = 28	Non-COVID-19 N = 27	*p*-Value
Eating 3 meals/day, n (%)			0.882
Daily	10 (35.7%)	8 (29.6%)	
Most of the days	10 (35.7%)	11 (40.7%)	
Having breakfast daily	14 (51.9%)	16 (59.3%)	0.584
Main meal of the day, n (%)			0.105
Breakfast	1 (3.6%)	6 (22.2%)	
Lunch	19 (67.9%)	16 (59.3%)	
Dinner	8 (28.6%)	5 (18.5%)	
Eating during night, n (%)	1 (3.6%)	0	-
Eating dinner after 21:00 daily or most of the times, n (%)	4 (14.8%)	4 (14.8%)	-
Eating while watching TV, n (%)	19 (67.9%)	15 (55.6%)	0.348

N/n (%) = number (percentage) of participants.

**Table 3:** Hunger, cognitive control of eating, ghrelin and NPY levels according to COVID-19 status.

	COVID-19 N = 28	Non-COVID-19 N = 27	*p*-Value
Hunger sensation	4.0 (2.0; 5.0)	5.0 (2.0; 7.5)	0.206
Fullness sensation	5.0 (3.0; 5.5)	5.0 (3.0; 5.5)	0.769
Desire to eat	1.0 (0.5; 3.0)	2.0 (1.0; 3.0)	0.477
Cognitive restraint	12.5 (9.5; 16.0)	12.0 (9.5; 13.0)	0.374
Uncontrolled eating	18.0 (15.5; 21.0)	16.0 (14.5; 19.0)	0.118
Emotional eating	6.0 (5.0; 7.0)	5.0 (3.0; 6.5)	0.102
Ghrelin, pg/mL	197.5 (121.3; 274.9)	67.1 (55.9; 80.4)	<0.001
NPY, pg/mL	128.0 (106.7; 164.6)	84.5 (67.9; 134.0)	0.005

NPY = neuropeptide Y; N/n (%) = number (percentage) of participants. Data in the table are provided as the median (quartile 1; quartile 3), unless otherwise specified.

**Table 4:** Brain regions significantly correlated with NPY.

Parameter	Brain Region with Clusters That Showed Significant Differences between Groups	Brodmann Area	Peak MNI Coordinates of the Cluster			Cluster Size (Voxels)
			x	y	z	
DC	Lingual, L	18 L	−15	−72	−6	102
ReHo	Lingual, L	37 L	−21	−42	−6	163

DC = degree of centrality; ReHo = regional homogeneity; L = left; MNI = Montreal Neurological Institute.

## Data Availability

Datasets are available on request from the authors.

## References

[1] Rothan H.A., Byrareddy S.N. (2020). The epidemiology and pathogenesis of coronavirus disease (COVID-19) outbreak. J. Autoimmun..

[2] Tang Y., Liu J., Zhang D., Xu Z., Ji J., Wen C. (2020). Cytokine Storm in COVID-19: The Current Evidence and Treatment Strategies. Front. Immunol..

[3] Qin C., Zhou L., Hu Z., Zhang S., Yang S., Tao Y., Xie C., Ma K., Shang K., Wang W. (2020). Dysregulation of Immune Response in Patients with Coronavirus 2019 (COVID-19) in Wuhan, China. Clin. Infect. Dis..

[4] Chen G., Wu D., Guo W., Cao Y., Huang D., Wang H., Wang T., Zhang X., Chen H., Yu H. (2020). Clinical and immunological features of severe and moderate coronavirus disease 2019. J. Clin. Investig..

[5] Chaaban N., Høier A.T.Z.B., Andersen B.V. (2021). A Detailed Characterisation of Appetite, Sensory Perceptional, and Eating-Behavioural Effects of COVID-19: Self-Reports from the Acute and Post-Acute Phase of Disease. Foods.

[6] Campos A., Port J.D., Acosta A. (2022). Integrative Hedonic and Homeostatic Food Intake Regulation by the Central Nervous System: Insights from Neuroimaging. Brain Sci..

[7] Müller T.D., Nogueiras R., Andermann M.L., Andrews Z.B., Anker S.D., Argente J., Batterham R.L., Benoit S.C., Bowers C.Y., Broglio F. (2015). Ghrelin. Mol. Metab..

[8] Mathur N., Mehdi S.F., Anipindi M., Aziz M., Khan S.A., Kondakindi H., Lowell B., Wang P., Roth J. (2021). Ghrelin as an Anti-Sepsis Peptide: Review. Front. Immunol..

[9] Kuliczkowska-Płaksej J., Jawiarczyk-Przybyłowska A., Zembska A., Kolačkov K., Syrycka J., Kałużny M., Polowczyk-Kawałko B., Kubicka E., Bolanowski M. (2023). Ghrelin and Leptin Concentrations in Patients after SARS-CoV2 Infection. J. Clin. Med..

[10] Ma Y., Zhang H., Guo W., Yu L. (2022). Potential role of ghrelin in the regulation of inflammation. FASEB J..

[11] Baatar D., Patel K., Taub D.D. (2011). The effects of ghrelin on inflammation and the immune system. Mol. Cell. Endocrinol..

[12] Yorulmaz H., Ozkok E., Ates G., Tamer S. (2017). Investigation of the effectiveness of ghrelin treatment in lung tissue of rats with sepsis. Bratisl. Lek. Listy.

[13] Hakami N.Y., Alhazmi W.A., Taibah E.O., Sindi M.M., Alotaibi O.F., Al-Otaibi H.M., Alhadrami H.A. (2021). The Effect of COVID-19 Infection on Human Blood Ghrelin Hormone: A Pilot Study. J. Pharm. Res. Int..

[14] Bakuradze T., Montoya Parra G.A., Riedel A., Somoza V., Lang R., Dieminger N., Hofmann T., Winkler S., Hassmann U., Marko D. (2014). Four-week coffee consumption affects energy intake, satiety regulation, body fat, and protects DNAintegrity. Food Res. Int..

[15] Muñoz J.S.G., Cañavate R., Hernández C.M., Cara-Salmerón V., Morante J.J.H. (2017). The association among chronotype, timing of food intake and food preferences depends on body mass status. Eur. J. Clin. Nutr..

[16] Yeomans M.R. (2010). Alcohol, appetite and energy balance: Is alcohol intake a risk factor for obesity?. Physiol. Behav..

[17] Spitzer R.L., Kroenke K., Williams J.B., Löwe B. (2006). A brief measure for assessing generalized anxiety disorder: The GAD-7. Arch. Intern. Med..

[18] Kroenke K., Spitzer R.L., Williams J.B. (2001). The PHQ-9: Validity of a brief depression severity measure. J. Gen. Intern. Med..

[19] Cotiga A.C., Zanfirescu S.A., Iliescu D., Ciumageanu M., Gotca I., Popa C.O. (2023). Psychometric Characteristics of the Romanian Adaptation of the GAD-7. J. Psychopathol. Behav. Assess..

[20] Murray A.L., Hemady C.L., Do H., Dunne M., Foley S., Osafo J., Sikander S., Madrid B., Baban A., Taut D. (2022). Measuring antenatal depressive symptoms across the world: A validation and cross-country invariance analysis of the Patient Health Questionnaire-9 (PHQ-9) in eight diverse low-resource settings. Psychol. Assess..

[21] Nechifor R.E., Ciobanu D., Vonica C.L., Popita C., Roman G., Bala C., Mocan A., Inceu G., Craciun A., Rusu A. (2020). Social jetlag and sleep deprivation are associated with altered activity in the reward-related brain areas: An exploratory resting-state fMRI study. Sleep Med..

[22] Flint A., Raben A., Blundell J.E., Astrup A. (2000). Reproducibility, power and validity of visual analogue scales in assessment of appetite sensations in single test meal studies. Int. J. Obes. Relat. Metab. Disord..

[23] Roman G., Bala C., Creteanu G., Graur M., Morosanu M., Popa A., Pircalaboiu L., Radulian G., Timar R., Achimas Cadariu A. (2015). Obesity and Health-Related Lifestyle Factors in the General Population in Romania: A Cross Sectional Study. Acta Endocrinol..

[24] Karlsson J., Persson L.O., Sjöström L., Sullivan M. (2000). Psychometric properties and factor structure of the Three-Factor Eating Questionnaire (TFEQ) in obese men and women. Results from the Swedish Obese Subjects (SOS) study. Int. J. Obes. Relat. Metab. Disord..

[25] Chao-Gan Y., Yu-Feng Z. (2010). DPARSF: A MATLAB Toolbox for “Pipeline” Data Analysis of Resting-State fMRI. Front. Syst. Neurosci..

[26] Yan C.G., Cheung B., Kelly C., Colcombe S., Craddock R.C., Di Martino A., Li Q., Zuo X.N., Castellanos F.X., Milham M.P. (2013). A comprehensive assessment of regional variation in the impact of head micromovements on functional connectomics. Neuroimage.

[27] Yan C.G., Craddock R.C., Zuo X.N., Zang Y.F., Milham M.P. (2013). Standardizing the intrinsic brain: Towards robust measurement of inter-individual variation in 1000 functional connectomes. Neuroimage.

[28] Ashburner J. (2007). A fast diffeomorphic image registration algorithm. Neuroimage.

[29] Jenkinson M., Bannister P., Brady M., Smith S. (2002). Improved optimization for the robust and accurate linear registration and motion correction of brain images. Neuroimage.

[30] Jiang L., Zuo X.N. (2016). Regional Homogeneity: A Multimodal, Multiscale Neuroimaging Marker of the Human Connectome. Neuroscientist.

[31] Zuo X.N., Ehmke R., Mennes M., Imperati D., Castellanos F.X., Sporns O., Milham M.P. (2012). Network centrality in the human functional connectome. Cereb. Cortex.

[32] Yan C.G., Wang X.D., Zuo X.N., Zang Y.F. (2016). DPABI: Data Processing & Analysis for (Resting-State) Brain Imaging. Neuroinformatics.

[33] Kojima M., Hosoda H., Date Y., Nakazato M., Matsuo H., Kangawa K. (1999). Ghrelin is a growth-hormone-releasing acylated peptide from stomach. Nature.

[34] Korbonits M., Goldstone A.P., Gueorguiev M., Grossman A.B. (2004). Ghrelin—A hormone with multiple functions. Front. Neuroendocrinol..

[35] Lee H.M., Wang G., Englander E.W., Kojima M., Greeley G.H. (2002). Ghrelin, a new gastrointestinal endocrine peptide that stimulates insulin secretion: Enteric distribution, ontogeny, influence of endocrine, and dietary manipulations. Endocrinology.

[36] Masuda Y., Tanaka T., Inomata N., Ohnuma N., Tanaka S., Itoh Z., Hosoda H., Kojima M., Kangawa K. (2000). Ghrelin stimulates gastric acid secretion and motility in rats. Biochem. Biophys. Res. Commun..

[37] Nagaya N., Kojima M., Uematsu M., Yamagishi M., Hosoda H., Oya H., Hayashi Y., Kangawa K. (2001). Hemodynamic and hormonal effects of human ghrelin in healthy volunteers. Am. J. Physiol. Regul. Integr. Comp. Physiol..

[38] Seminara R.S., Jeet C., Biswas S., Kanwal B., Iftikhar W., Sakibuzzaman M., Rutkofsky I.H. (2018). The Neurocognitive Effects of Ghrelin-induced Signaling on the Hippocampus: A Promising Approach to Alzheimer’s Disease. Cureus.

[39] Dixit V.D., Schaffer E.M., Pyle R.S., Collins G.D., Sakthivel S.K., Palaniappan R., Lillard J.W., Taub D.D. (2004). Ghrelin inhibits leptin- and activation-induced proinflammatory cytokine expression by human monocytes and T cells. J. Clin. Investig..

[40] Sato T., Nakamura Y., Shiimura Y., Ohgusu H., Kangawa K., Kojima M. (2012). Structure, regulation and function of ghrelin. J. Biochem..

[41] Tanaka M., Yamada S., Watanabe Y. (2021). The Role of Neuropeptide Y in the Nucleus Accumbens. Int. J. Mol. Sci..

[42] Yi M., Li H., Wu Z., Yan J., Liu Q., Ou C., Chen M. (2018). A Promising Therapeutic Target for Metabolic Diseases: Neuropeptide Y Receptors in Humans. Cell. Physiol. Biochem..

[43] Beck B. (2006). Neuropeptide Y in normal eating and in genetic and dietary-induced obesity. Philos. Trans. R. Soc. Lond. B Biol. Sci..

[44] Kalra S.P., Kalra P.S. (2003). Neuropeptide Y. A Physiological Orexigen Modulated by the Feedback Action of Ghrelin and Leptin. Endocrine.

[45] van Meer F., van der Laan L.N., Adan R.A., Viergever M.A., Smeets P.A. (2015). What you see is what you eat: An ALE meta-analysis of the neural correlates of food viewing in children and adolescents. Neuroimage.

[46] Little T.J., McKie S., Jones R.B., D’Amato M., Smith C., Kiss O., Thompson D.G., McLaughlin J.T. (2014). Mapping glucose-mediated gut-to-brain signalling pathways in humans. Neuroimage.

[47] Stopyra M.A., Friederich H.C., Lavandier N., Mönning E., Bendszus M., Herzog W., Simon J.J. (2021). Homeostasis and food craving in obesity: A functional MRI study. Int. J. Obes..

